# Therapy of Non-Dermatophytic Mycoses in Animals

**DOI:** 10.3390/jof4040120

**Published:** 2018-10-30

**Authors:** Daniel Elad

**Affiliations:** Department of Clinical Bacteriology and Mycology, The Kimron Veterinary Institute, P.O. Box 12, Bet Dagan 50250, Israel; daniel.elad@gmail.com; Tel.: +972-3-968168; Fax: +972-3-9681733

**Keywords:** animal, mycosis, therapy, disseminated, upper respiratory tract, dimorphic, eumycetoma, cryptococcosis, *Malassezia*

## Abstract

This review focuses on aspects of antimycotic therapy specific to veterinary medicine. In the first part, drug availability, limited mostly by economic consideration but also by clinical applicability and specific adverse effects, is described for polyenes, 5 fluorocytosine, azoles, echinocandins and terbinafine. In the second part, current knowledge and experience in the treatment of selected fungal infections are overviewed. These mycoses include disseminated mold infections in small animals (dogs and cats) and avian species, upper respiratory tract infections of small animals (sino-nasal and sino-orbital aspergillosis) and horses (guttural pouch mycosis), eumycetoma, infections caused by dimorphic fungi, (blastomycosis, histoplasmosis, coccidioidomycosis, paracoccidioidomycosis and sporothrichosis) and by yeasts and yeast-like microorganism (*Cryptococcus* spp. and *Malassezia pachydermatis*).

## 1. Introduction

Veterinary medical mycology often differs from the human counterpart by, among others, the clinical aspects (beyond the scope of this review), the variety of fungi involved and the antimycotic drugs available for use. Immunosuppression, induced by other infective agents or by medications, has led to a significant leap in the number of people affected and the species of fungi involved in human infections since the 1980s [1]. In veterinary mycology, this phenomenon was not observed. Thus, changes in the range of fungi involved in animal mycoses stems mostly from emergence of new pathogens such as *Cryptococcus gattii* or *Sporothrix brasiliensis*, increased awareness, or changes in taxonomy due to improved molecular techniques such as has happened for the *Pseudallescheria/Scedosporium/Lomentospora* group [2].

The incentive to develop new antimycotic drugs for human medicine, having better pharmacological characteristics, a broader spectrum and fewer side effects, was driven to a large extent by the aforementioned surge in mycotic infections’ number and the variety of the etiological agents involved. Some of these newly developed antimycotic drugs (and a few older ones) are exceedingly expensive (detailed below). Economic considerations have a much more significant impact on the decision to treat for animals than for humans and are based primarily on the value of the animal (i.e., for breeding). In addition, the sentimental implications of euthanasia as an alternative to treatment are of significance, especially for pets [3]. The importance of the drug cost is further emphasized by the fact that in many cases the infections relapsed after the animals have seemingly recovered and were refractory to further treatments (detailed below). Thus, a prolonged, possibly lifelong, administration of the drug may be necessary. Other differences from human medicine result from practical considerations (i.e., the stress resulting from repeated drug administration to wild animals) and specific adverse effects.

Fungal susceptibility testing has been standardized [4] for human isolates. Due to anatomical and physiological differences between people and animals, the predictive value of such tests for the latter has not been demonstrated, and thus their use for veterinary fungal infections is questionable.

One of the outcomes of taxonomic changes in the classification of certain fungi and their division into several species is their different susceptibility to antimycotic drugs, as exemplified by the *Pseudallescheria/Scedosporium/Lomentospora* group [5] or *Aspergillus fumigatus*/*A. felis* (detailed below). Consequently, the likelihood of a successful therapy depends on the accurate identification of the fungus involved in the infection.

Since this review is aimed at providing a veterinary perspective on antifungal therapy and not to provide guidelines, periods of treatments are selectively included whereas dosages are not.

## 2. Systemic Antimycotic Drugs Currently Available for Veterinary Use and Their Side Effects

### 2.1. Amphotericin B (AmB)

AmB is a polyene antimycotic drug that was discovered more than 6 decades ago. Nevertheless, AmB (colloidal dispersion in sodium deoxycholate) still acts as a reference for other, newer, compounds, mostly due to its broad spectrum of action [6]. Unfortunately, one of the main etiological agents of canine disseminated aspergillosis, *A. terreus*, is relatively resistant to this drug [7]. Since it is not absorbed from the gastrointestinal system, it must be administered intravenously (IV), causing inherent problems in treating animals with the drug. Novel approaches aimed at improving the oral availability of AmB by using various excipients have been published [8].

A few attempts to administer AmB subcutaneously resulted in adverse local reactions in some cases [9]. The main, but not only, side effect of AmB is its marked nephrotoxicity. To obviate to this, variants have been developed, namely a liposomal preparation, a lipid complex and a colloidal dispersion in cholesterol sulphate. Although significantly less toxic, these preparations are also considerably more expensive and thus have limited use in veterinary medicine. Consequently, AmB is used in veterinary medicine mostly in cases in which other, less problematic, drugs fail [10].

Two other polyene antimycotic drugs, used in topical treatments, are nystatin and natamycin. The latter is the mainstay of keratomycosis treatment in human and veterinary medicine [11].

### 2.2. 5 Fluorocytosine (5FC)

5FC inhibits fungal nucleic acid synthesis. Resistance to 5FC emerges quickly and thus it is unsuitable for monotherapy. It is synergic with AmB and their combination was, before the discovery of newer drugs such as fluconazole, the choice for the treatment of, primarily, cryptococcosis and *Candida* spp. infections [12].

Side effects in dogs may include bone marrow suppression, gastrointestinal lesions and neurotoxicity. Cutaneous and muco-cutaneous ulcerations were associated in 7 of 8 dogs treated with the drug. The lesions continued to develop for up to a month following therapy cessation and took about 2 weeks to heal [13].

### 2.3. Azoles

Azoles comprise topical and systemic drugs. The first of the latter was ketoconazole (KCZ), an imidazole, originally used in veterinary medicine to treat various cutaneous and systemic mycoses. Adverse effects include gastrointestinal disturbances, hepatotoxicity, possible inhibition of testosterone production, and interaction with the metabolism of other drugs. It has been superseded by the triazoles fluconazole (FCZ) and itraconazole (ICZ), both less toxic and with better therapeutic and pharmacodynamic and pharmacokinetic characteristics.

The most significant advantage of FCZ is its ability (shared neither by KCZ nor ICZ) to pass the barriers to the brain, the eye and the prostate, thus making it suitable to treat mycotic infections of these organs. Side effects of FCZ may include gastrointestinal disturbances and hepatotoxicity and it is considered teratogenic. More rarely, FCZ may induce polyuria/polydipsia and hair loss [9].

ICZ is the drug of choice for the treatment of systemic infections in small animals. Although unable to penetrate the anatomic barriers as efficiently as FCZ, the inflammatory processes in the relevant organs may enable the drug to reach therapeutic levels. Adverse effects include gastrointestinal disturbances, hepatotoxicity and interaction with the metabolism of other drugs. In addition, suppurative skin lesions, resulting from vasculitis have been reported in dogs, especially when treated with higher doses of the drug [14].

The first of a second generation of triazoles were voriconazole (VCZ) and posaconazole (PCZ). Although the former is off patent, and the latter will follow in 2019, their prices are still too high to be adopted on a large scale in veterinary medicine and consequently experience with these drugs is limited. A preparation for the treatment of canine otitis that contains PCZ (Posatextm, Merck) and an ophthalmic ointment containing VCZ (Wedgewood Pharmacy) are, however, available.

The side effects of VCZ include drug interaction, teratogenicity [10] and visual disorders and neurotoxicity in cats [15]. Veterinary experience with PCZ is too limited to delineate specific animal adverse effects, but drug interactions have been reported in human patients [16].

An additional imidazole, enilconazole (ECZ), has been specifically developed for veterinary use for environmental antifungal disinfection by fumigation or topical spray application, useful in upper respiratory tract mycoses [11].

### 2.4. Echinocandins

Echinocandins inhibit glucan synthesis and since this mechanism of action differs from that of other antimycotic drugs, cross-resistance is unlikely. The first drug in this group to be commercialized was caspofungin (CFG), active primarily against *Aspergillus* spp. and *Candida* spp. The administration is IV only. CFG is off patent and other echinocandins (micafungin and anidulafungin) will follow in the next 2–3 years.

The limited veterinary experience with CFG does not permit specific animal adverse effects of the drug to be outlined, but interactions have been reported in human patients [17].

### 2.5. Terbinafine (TBF)

TBF is an allylamine with high affinity to the skin and its adnexa and is, consequently, used to treat dermatomycoses, replacing griseofulvin. It was found, however, to be useful, often in combination with other drugs, in treating a variety of mycoses (detailed below).

## 3. Molds

### 3.1. Disseminated Mycoses

#### 3.1.1. Dogs

These infections are arguably among the most frustrating. They are difficult to diagnose, unless suspected and appropriate tests such as microscopy of biopsies and/or urine are performed and show the tell-tale hyphae [18]. Consequently, affected animals are often treated for other ailments before diagnosis, sometimes with steroids, which may facilitate the fungus’ dissemination to organs. Thus, by the time the correct diagnosis is made the infection has spread to various organs such as bones/joints, the kidney and the brain [19]. Moreover, one of the most common fungi involved in disseminated mold infections, especially in German Shepherd dogs, is *A. terreus* [20]. This fungus is notoriously resistant to antimycotic therapy, possibly because of the low ergosterol (a target to several important antimycotic drug classes) content in its cell membrane. All these factors lead to an extremely poor prognosis.

Attempts at treating disseminated mold infections are presented in Table 1. Only a fraction of these attempts showed some success, whereas the large majority failed, often with relapses after initial improvement, the owners’ refusal to treat after periods of varying length or being lost to follow-up. In fact, out of 59 cases in which therapy was attempted for more than 7 days, success was reported in only 7 (Table 1).

#### 3.1.2. Avian Species

Another group of animals that is affected by disseminated mold infections are avian species. The systemic dissemination of molds in these animals is expedited by the presence of coelomic cavities (“air sacs”) that expand from the lungs to various organs including the bones [60]. *Aspergillus* spp. are the molds most frequently involved. Infections in large groups are observed in commercial enterprises and are caused primarily by environmental contamination, especially of the feed. Consequently, improved management and environmental decontamination are the conventional methods to deal with such infections. When individual birds are affected, a different approach is necessary. Factors such as the stress induced by repeated capture needed for treatment must be considered [61]. In addition, pharmacokinetic aspects of the drugs administered must be taken into consideration to ensure suitable concentrations are achieved in the affected organs [62]. Antimycotic therapy by oral administration is preferred and nebulization with nanosuspensions of ICZ have been attempted [63,64]. VCZ has been used in several studies in wild birds, including African penguins [65] and red-tailed hawks [66]. Pigeons experimentally infected with *A. fumigatus* were successfully treated with VCZ [67]. A 70% cure rate in naturally infected falcons was achieved with the same drug [68]. Silvanose et al. [69] found that while treating *Aspergillus* spp. in falcons with AmB, ITC and VCZ, the MIC increased for AmB but not for the two azoles. It should be stressed that treatment methods for birds have not been standardized and thus care should be taken especially since long periods of drug administration may be necessary [70].

### 3.2. Upper Respiratory Tract Mycoses

The most common fungal infection of the upper respiratory tract (URT) involves the sino-nasal (SN) cavities and predominantly affects dogs. Cranial conformation is considered a predisposing factor with an overrepresentation of dolichocephalic and mesocephalic dogs [71]. The most common etiologic agent is *A. fumigatus* [72]. Systemic treatment with thiabendazole (TBZ), KCZ, FCZ or ICZ resulted in success rates of about 50% for the former two and around 70% for the latter two [71]. Since the fungi are not invasive, their contact with systemic antimycotic drugs alone may not suffice to treat the infection. This may be obviated by supplementing the systemic treatment with a topical one administered by catheters or following trephination of the frontal sinuses. Clotrimazole (CTZ) or ECZ have been reported to have success rates of 90% and above. In cases in which the cribriform plate’s integrity has been compromised, the drugs may reach the brain and consequently topical treatments are contraindicated [71]. A cat with SN mycosis caused by *A. fumigatus*, was successfully treated with debridement and PCZ, replaced with ICZ after relapse [73].

In cats, the most frequent URT mycosis consists of sino-orbital (SO) infection. Brachycephalic cats are at a higher risk [74]. SO mycoses are caused primarily by *Aspergillus felis* (*Neosartorya* morph). This fungus is both more invasive and more resistant to antimycotic drugs than *A. fumigatus* [75,76]. This invasiveness and the anatomical localization of the retroorbital cavities above the nasal cavities may be the reason for the evolution of SN mycoses to SO infections.

Barrs et al. [74] attempted to treat 6 cases of feline sino-nasal and 15 cases of SO aspergilloses. Cats were treated with AmB and ICZ or PCZ (5 cats), AmB (deoxycholate or liposomal) and ICZ or PCZ and TBF (7 cats) and 14 cats were treated with ICZ, PCZ or VCZ (2, 8 and 4 cats, respectively). Four cats with SN aspergillosis and one with SO aspergillosis were successfully cured. The former were treated with (a) deoxycholate AmB and ICZ or FCZ, and TBF, (b) deoxycholate AmB, liposomal AmB, PCZ and TBF, (c) deoxycholate AmB and TBF, (d) PCZ. One cat affected by SO aspergillosis recovered following treatment with deoxycholate AmB and ICZ. Initial cure was followed by a relapse that was treated with PCZ and TBF for 32 and 16 weeks, respectively. However, the cat relapsed again and was treated unsuccessfully with liposomal AmB and PCZ. Treatment with CFG followed by PCZ finally led to the cat’s recovery. The variability of the successful protocols and the fact that similar protocols applied to other cats failed indicate that no single treatment method of feline URT mycotic infection may be recommended.

### 3.3. Guttural Pouch Mycosis (GPM)

The guttural pouch is a diverticulum of the Eustachian tubes present in several animal groups. Among domestic animals, horses are the only ones to have this organ [77]. The fungi involved are primarily *A. fumigatus* and *A.* (*Emericella*) *nidulans* [78].

Since the GP is in contact with some major arteries and nerves, the infection may be complicated by severe, potentially life-threatening epistaxis and/or dysphagia [78]. The former may be treated/prevented by surgical procedures. For the latter, functional recovery, and thus survival, is rare [78,79].

Prior to the introduction of systemic azoles, GPM antimycotic therapy was based on the topical application of natamycin, iodine preparations, nystatin and oral or topical TBZ or abendazole. Powdered preparations had better contact with the fungal plaque than liquid ones and, thus, were more effective [79]. The topical infusion and oral paste of ICZ was found to be effective in one case [78].

Five cases of GPM without epistaxis but with dysphagia were treated with topical natamycin [80]. This treatment resulted in no clinical improvement in three horses and one horse made a partial recovery. There was no follow-up for the fifth horse, but the referring veterinarian and the owner reported no respiratory impediment. An additional case, using more contemporary drugs, [81] treated the fungal infection successfully with ICZ and topical ECZ. After several weeks, the swallowing problems of the horse disappeared as well.

### 3.4. Eumycetoma

Eumycetoma are subcutaneous or visceral mycoses characterized by a chronic course, tumefaction, and “granules” consisting of fungal aggregates that are secreted from the lesions through sinuses [82]. In human cases, surgery in combination with ICZ is the most common treatment, but a few studies indicate that PCZ and TBF alone or in combination with ICZ, may be effective [83].

These mycoses are reported rarely from animals and information on therapeutic attempts is scant. In one case [84], caused by *Curvularia lunata*, the tumefaction has invaded the dog’s inguinal muscular tissue and could not be fully excised. The dog was treated for 3 months with ICZ following which the tumefaction regressed completely. The therapy was continued for 2 additional months, but 4 months after its cessation the inguinal tumefaction reappeared and was refractory to further treatment with ICZ. Susceptibility discs were not available at the time of the initial isolation but became obtainable by the time of the relapse. Thus, susceptibility was tested only for the latter isolate resulting in an inhibition zone of 30 mm, probably indicating that, contrary to the in vivo lack of activity, in vitro the fungus was still susceptible to the drug. More recently, Janovec et al. [85] reported the successful treatment with itraconazole of a dog with visceral mycetoma caused by *Penicillium duponti.*

### 3.5. Keratomycoses

Keratomycoses are fungal infections of the eyes of various animals, most frequently horses (recently reviewed by Ledbetter [86]). Keratomycosis develops most frequently following the traumatic implantation of the infecting microorganism. Horses’ protruding eyes and lateral localization may be a risk factor that contributes to their predisposition to these infections [87]. Although various fungal species have been isolated from cases of equine keratomycosis [86], the most prevalent etiology is *Aspergillus* spp., especially *A. fumigatus* [88].

Antimycotic therapy may be topical or systemic. Topical drugs include primarily azoles (miconazole, itraconazole, fluconazole and voriconazole) and polyenes (natamycin, nystatin and amphotericin B). Following the systemic administration of antifungal drugs, fluconazole and voriconazole but not itraconazole have been found to reach therapeutic levels in the aqueous humor [86]. Although antimycotic therapy may suffice to resolve some cases of equine fungal eye infections, surgical enucleation or exenteration may be necessary in others [87].

## 4. Dimorphic Fungi

Dimorphic fungi are characterized by a saprophytic, environmental, mold phase and a parasitic yeast phase in the host. Exposure occurs mostly by inhalation of conidia produced by the saprophytic phase. In the lungs, conidia develop into yeast cells that may remain localized or disseminate to various organs. In most cases, no clinical signs develop. Since the transmission of yeast cells is exceptional (one case of a veterinarian bitten by a dog with blastomycosis has been reported [89]) these mycoses are considered neither contagious nor zoonotic.

### 4.1. Blastomycosis

Blastomycosis is caused by *Blastomyces dermatitidis* and is endemic to North America, but has been reported from other parts of the world as well [90]. Treatment with AmB followed by KCZ or, preferably, ICZ or with ICZ alone are recommended in severe and lighter cases, respectively [91]. The treatment’s duration may vary but should be continued for 1–2 months after clinical and radiographic recovery and is successful in 70–80% of the cases but relapses may occur. About half of 112 canine cases of blastomycosis were cured with ICZ [14]. Among the dogs that died or relapsed, central nervous system (CNS) involvement was overrepresented. FCZ, although less effective, may be recommended in cases of urinary tract and CNS infections due to this drug’s ability to penetrate these compartments [92]. Among the newer triazoles, VCZ and PCZ show promise, but experience with these drugs in animals clinically infected is still too scant to reach any conclusions.

### 4.2. Paracoccidioidomycosis

This mycosis, caused by *Paracoccidioides brasiliensis*, is endemic in South America and only rarely causes clinical infections in animals. Seroconversion, however, was reported with a significantly higher prevalence in rural dogs [93]. Gonzales et al. [94] report a case of paracoccidioidomycosis of a cat, involving the brain and the urinary tract. Diagnosis was based on the presence of typical fungal elements in the urine. No potentially immunosuppressive predisposing factors were found. The cat was treated with a single administration of FCZ IV. After short periods of oral KCZ or ICZ causing adverse reactions or being inefficient, respectively, the cat was treated with FCZ for 3 years with the supplement of AmB for 12 weeks during the second year, with no adverse effects. Subsequently, the dose of FCZ was reduced and finally stopped. The cat showed no clinical signs and no fungal elements in the urine and was eventually euthanized after 5 years for seemingly unrelated reasons (uremic syndrome).

### 4.3. Histoplasmosis

Histoplasmosis is caused by *Histoplasma capsulatum* (except for Africa where *H. duboisii* is endemic) and has a worldwide distribution. The fungus has a large prevalence in the eastern parts of North America where it frequently affects dogs and cats [95]. The treatment of choice is ICZ for 4 to 6 months. Although relapses may occur, further treatment was successful in treating the animals [95]. FCZ may be used if the brain is involved. In cases of ICZ treatment failures, AmB may be considered despite its toxicity.

### 4.4. Coccidioidomycosis

Coccidioidomycosis is caused by *Coccidioides immitis* endemic in California and *C. posadasii*, endemic primarily in the semiarid lower Sonoran region that extends on both sides of the United States–Mexican border and some areas of Central and South America [96]. Concerns as to the possible spread of endemic zones due to climate changes have been expressed [97].

The fungus infects a wide variety of mammals, including marine species, and some reptiles [9]. Among small animals, dogs undergo more frequently asymptomatic seroconversion (about 70%) than cats, but the latter seem to develop more severe, systemic, infections [9]. Clinical symptoms result from pulmonary involvement and, in cases of dissemination, from organs affected such as the bones or the CNS. Cutaneous lesions are more common in cats than in dogs. Epi-, peri- and myocardium may be affected and lead to sudden death [98].

FCZ is the drug of choice for the treatment of coccidioidomycosis in animals. AmB may be used in severe cases or those in which azole therapy fails [9]. An attempt to treat a dog with FCZ and AmB subcutaneously resulted in a local reaction that necessitated the termination of the process [9]. Shultz et al. [99] used a novel antimycotic drug, nikkomycin Z, that inhibits chitin synthesis with promising results: out of 9 dogs with pneumonic coccidioidomycosis that were treated for 60 days, 3 and 4 showed marked and partial improvement, respectively.

### 4.5. Sporotrichosis

Sporothrichosis is a subcutaneous mycosis that may be restricted to the skin or disseminate to various organs [100], The most prevalent etiologic agents are the dimorphic fungi *Sporothrix shenkii*, *S. globosa* or *S. brasiliensis*. While the former two have worldwide distribution, the latter is endemic in Brazil, where it is a major cause of feline sporothrichosis. Various animals may be affected, but cats seem to be the most prevalent host, carrying a large number of yeast cells in their lesions, mouth and claws, thus posing a significant zoonotic threat.

Drugs used to treat sporothrichosis include potassium iodide (that may cause severe adverse effects in cats), ICZ and/or TBF. The administration should continue at least for one month after clinical cure [101]. Cases refractory to these drugs may be treated with AmB, with intra-lesional administration being more successful and having less adverse side effects. All these therapeutic approaches have, however, a high rate of failures or relapses [102]. Non-pharmacological treatment methods include surgical removal of lesions and thermotherapy [102].

Recently, de Miranda et al. [100] reported treatment with ICZ alone (74 cats) or in combination with potassium iodide (56 cats). While 14 cats treated with the monotherapy remained positive, all the animals treated with the combination recovered. Cats that were refractory to treatment had a higher initial yeast count in the lesions. In the recovered cats, the microscopic fungal counts decreased to zero after 12 weeks, thus reducing significantly the risk of the fungus’ spread. Recently, two dogs were treated successfully with terbinafine monotherapy [103].

In addition to antifungal drugs, pentamidine (an anti-Leishmania compound) showed anti-*Sporothrix* activity and synergism with AmB, ICZ and TBF [104].

## 5. Yeasts and Yeast-Like Fungi

### 5.1. Cryptococcosis

Cryptococcosis is caused in animals and humans primarily by two basidiomycetous, encapsulated yeast species: *Cryptococcus neoformans* and *C. gattii*. The environmental niche of the two species differs: while *C. neoformans* is found mostly in bird droppings, *C. gattii* is associated with trees [105].

Animal cryptococcosis does not seem to be associated with immune deficiencies [106]. Among animals, cats are the most frequently affected, but cases in a large variety of animals, including dogs, horses, koalas and marine mammals have been reported [107]. Various organs may be involved, but the central nervous system or the upper respiratory apparatus is affected most frequently in dogs and cats, respectively. The latter may develop subcutaneous granulomata [108].

Therapy of small animals (dogs and cats) is based on AmB with or without 5FC (may cause severe adverse reactions in dogs [12]), ICZ, FCZ and TBF. Among these, FCZ is preferable in cases of CNS involvement due to its ability to cross the blood brain barrier. Malik et al, [13] developed a method of subcutaneous administration of AmB with a significant reduction of adverse effects, in addition to facilitating the administration procedure. One of three dogs treated by this method developed a dose-dependent cutaneous reaction. Several cases of gastrointestinal lesions were reported [109,110,111]. One of these cases [110] recovered following treatment based on AmB with 5FC, FCZ or ICZ administered for different periods. Another case [111], treated with AmB and FCZ improved clinically but remained culture positive.

Differences in the susceptibility of the various *Cryptococcus* spp. and genotypes to antimycotic drugs in general and FCZ in particular have been reported [112]. Consequently, the molecular identification of the isolates is important in defining the treatment protocol. Moreover, isolates with different geographical origins may show different susceptibilities [113].

O’Brien et al. [106] compared the efficacy of treatments with AmB, ICZ and FCZ in 59 cats and 11 dogs. The outcome was considered successful when antigenemia disappeared or was steadily reduced after therapy cessation, clinical signs were absent for at least 2 years after therapy termination or when clinical control could be obtained with long-term antimycotic therapy. Forty-five cats and 6 dogs fitted at least one of these criteria. No significant differences were found between the efficacy of the drugs for the cats, whereas AmB was included in the successful treatment protocols of the dogs.

Cryptococcosis in horses involves mostly the lower respiratory tract [114]. One case [115] of sino-nasal cryptococcosis was successfully treated by surgical removal of the granuloma, the local instillation of ECZ, and the systemic administration of FCZ. Another equine case [116] in which the CNS and optic nerve were affected, was treated successfully with oral FCZ for 197 days. Four horses with pulmonary cryptococcosis caused by *C. gattii* were treated successfully with oral administration of FCZ for 18, 7, 9 and 3 months, respectively [117].

### 5.2. Malassezia pachydermatis

The basidiomycetous genus *Malassezia* comprises an increasing number of lipophilic species, four of which have been associated with domestic animals [118]. Most commonly involved in animal infections is, however, *M. pachydermatis*, a species that does not require fatty acids for growth. It is a saprophyte of the skin of a variety of animals and an opportunistic pathogen involved primarily in canine otitis and dermatitis [119]. Various risk factors for the development of infections, including antibacterial and corticosteroid therapies, have been suggested [118].

Several drugs for the specific treatment of canine otitis that combine antibacterial, antimycotic and anti-inflammatory drugs, are commercially available. These drugs are aimed at the prevention of the proliferation of the bacterial or the mycotic components of the ear microbial flora that may occur under monotherapy. In an evidence-based review, Negre et al. [120] found that a combination of chlorhexidine and miconazole are recommended for the topical treatment of canine *Malassezia* dermatitis whereas itraconazole is the most suitable drug for systemic administration.

## 6. Conclusions

An ongoing trend of great advances in antifungal therapy during the last years characterizes human medicine. Unfortunately for the veterinary community, many of the newer drugs are too expensive to be broadly adopted to treat animal mycoses. Some antimycotic drugs that have come off patent are currently more accessible, but are still not an option for the often-necessary prolonged therapy courses. In addition, since several of these infections, such as disseminated mold infections in dogs, are rare compared to similar human infections and treatment protocols vary greatly, the adoption of a standard one is difficult.

Apparently, the increased importance of human mycoses and the resulting publication of research and clinical data have had a bearing on veterinary medicine. Additional information stemming from this trend will, hopefully, improve the success rate in the treatment of animal mycoses.

## Figures and Tables

**Table 1:** Etiology, therapy protocols and outcome of disseminated fungal infections in dogs.

Etiology	Therapy Protocol	Outcome	Ref.
*Aspergillus terreus*	AmB methyl ester + 5FC	Failure	[21]
*Aspergillus deflectus*	KCZ 4 m	Recovery after affected leg amputation	[22]
*Paecilomyces* spp.	KCZ 3 w	Failure. No autopsy	[23]
*Lomentospora prolificans*	ICZ 4 m	Failure	[24]
*Aspergillus terreus*	ICZ 2 m	Failure	[25]
*Acremonium* spp.	KCZ + ICZ 7 m	Failure	[26]
*Aspergillus terreus*	Hamycin	Failure	[27]
*Aspergillus terreus*	ICZ 33 m	Eut. 572 days after treatment cessation.	[28]
*Aspergillus terreus*	ICZ 6 m	Failure.	
*Aspergillus terreus*	ICZ 3 y	Eut. 485 d after treatment cessation	
*Aspergillus terreus*	ICZ 17 m	Recovery. Eut. after 3 y (heart failure). No fungi found at autopsy	
Case cohort. No association between aetiology and individual cases possible.	KCZ 2 w ICZ 11 w	Improvement. Eut. required by owners after 16 months, 12 of which without therapy	[29]
	KCZ 20 d ICZ 10 w	Improvement. Eut. required by owners	
	KCZ 1 w ICZ 21 m irregularly	Improvement. Eut. (lymphocytic leukaemia)	
	ICZ	Alive after 4 years	
*Paecylomyces* spp.	AMB + (KCZ then FCZ)	Improvement except eye then relapse 7 w Eut.	[30]
*Paecylomyces* spp.	5FC + FCZ	Eut.	[31]
*Paecilomyces* spp. ^1^	KCZ 4 m	Failure. Sudden death after apparent improvement.	[32]
*Phialemonium obovatum*	AmB 8 days ICZ 1 m KCZ 2 m ICZ	Failure. Eut. 5 m after initial evaluation.	[33]
*Paecilomyces variotii*	KCZ 6 m	Relapse 3 m after presentation. Eut..	[34]
*Cladophialophora bantiana*	FCZ 6 w	Eut.	[35]
*Schizophyllum commune*	KCZ 3 m	Failure	[36]
*Monocillium indicum*	ICZ 2 m	Relapse 7 m after presentation. Eut.	[37]
*Geomyces spp.*	ICZ, 6 m + 4 m	Clinical improvement	[38]
*Paecylomyces varioti*	ICZ, AmBLC 5 doses	Eut.	[39]
*Aspergillus terreus*	AmB Lipid Complex + ICZ	Failure. Eut.	[40]
?	ABLC ICZ	Failure. lost	
*Aspergillus niger*	ABLC + ICZ, ICZ + TBF	Failure. lost	
*Aspergillus deflectus*	ABLC + ICZ, deoxycholate AmB, ABLC + VCZ 7w, TBF + PCZ + ABLC, CFG 6 m, anidulafungin, ABLC + micafungin	Failure after 25 m	
*Aspergillus terreus*	Hemilaminectomy, VCZ 6 m PCZ 4 m	Failure, Eut. 13 m	
*Oxyporus corticola*	ICZ 6 m, TBF 10 m, ICZ 8 m	Eut.	[41]
*Pseudallescheria boydii*	ICZ 3 w	Failed. Eut.	[42]
*Aspergillus* spp.	FCZ 6 m	Failure. Eut.	[43]
*Phialosimplex caninus*	ICZ 2 w AmB lipid complex 6 d ICZ 17 m	Death due to unknown cause. No autopsy.	[44]
*Oxyporus corticola*	ICZ 18 d	Eut.	[45]
*Westerdykella* spp.	ICZ, LipAmB, PCZ 3 m, TBF 4 w	Eut.	[46]
*Plectosphaerella cucumerina Lecythophora canina*	ICZ+TBF	14 m after presentation improvement except visual impairment	[47]
*Phialosimplex caninus*	ICZ > 18 m	Recovered.	[48]
*Lomentospora prolificans*	ICZ + TBF 16 d VCZ + TBF 6 w	Improved but relapsed after 6 m Eut.	[49]
*Spiromastix asexualis*	ICZ	Eut.	[50]
*Scytalidium* spp.	ICZ 3 m	Failure. Eut.	[51]
*Chrysosporium* spp.	PCZ 36 d ICZ 11 w	Improved but relapsed 12 m later. Eut..	[52]
*Aspergillus terreus*	VCZ + TBF 11m + AmB 8 treatments	Alive with tetraparesis and, head tilt after 11 m	[20]
	FCZ 9 d VCZ + TBF	Lost to follow up	
*Geosmithia argillacea*	FCZ 10 m	Failure. Eut.	[53]
*Acremonium* spp.	ICZ 2 w, 2 w pause, 11 d	Eut.	[54]
Cohort of 10 dogs. No association between aetiology and individual cases possible. *A. terreus* × 5 *A. fumigatus* × 1 *A. veriscolor* × 1 *Aspergillus* spp. × 3	PCZ PCZ + TBF 15 m	9 dogs: survival 44–516 d 6 dogs: failure. Relapse at various times. 3 showed clinical improvement. 2 dogs relapsed after therapy cessation and 1 dog lost to follow-up 1 dog alive at 5 y	[55]
*Bipolaris spicifera*	FCZ 1 w, ICZ + TBF, LipAmB 1 d	Eut.	[56]
*Phanerochaete chrysosporium*	ICZ 7 m	Eut.	[57]
*Cladosporium cladosporioides*-*complex*	ICZ 5 m	Relapse after 25 m. Eut.	[58]

^1^ Subsequently identified as *Sagenomella chlamydospora* [59] and later reclassified as *Phialosimplex chlamydosporus* [48]. AmB: amphotericin B, KCZ: ketoconazole, ICZ: itraconazole, FCZ: fluconazole, VCZ: voriconazole, PCZ: posaconazole, CFG: caspofungin, TBF: terbinafine, AmBLC: amphotericin B lipid complex, LipAmB: liposomal amphotericin B, d: day, w: week, m: month, y: year, Eut: euthanasia, ?: unknown. Red font: successful treatment.
